# Association of Natriuretic Peptide With Adverse Outcomes and Disease Severity After Intracerebral Hemorrhage: A Systematic Review

**DOI:** 10.3389/fneur.2021.775085

**Published:** 2021-11-15

**Authors:** Jiahui Wang, Jingxuan Wang, Zhouping Tang, Ping Zhang

**Affiliations:** Department of Neurology, Tongji Hospital, Tongji Medical College, Huazhong University of Science and Technology, Wuhan, China

**Keywords:** intracerebral hemorrhage, brain natriuretic peptide, N-terminal pro-brain natriuretic peptide, prognosis, disease severity

## Abstract

**Background:** Over the past decade, many studies have reported the association of brain natriuretic peptide (BNP) and N-terminal pro-brain natriuretic peptide (NT-proBNP) with clinical outcome of intracerebral hemorrhage (ICH). However, a broad consensus has not been reached.

**Objective:** To evaluate the role of BNP/NT-proBNP levels in prognosis and disease severity assessment in patients with ICH.

**Methods:** A systematic literature search was conducted utilizing PubMed, Embase, Web of Science and the Cochrane Library databases up to July 23, 2021. Studies that explored the association between BNP/NT-proBNP level and clinical outcome or disease severity in ICH patients were eligible. Outcome measures were all-cause mortality, poor functional outcome, adverse cardiac events and markers of disease severity.

**Results:** Ten studies, involving 1,373 patients with ICH, met the inclusion criteria. Nine studies focused on clinical outcomes (five all-cause mortality, five functional outcomes, and one adverse cardiac event) and seven on disease severity. In terms of prognosis, all five studies showed an association between elevated BNP/NT-proBNP level and increased risk of all-cause mortality in ICH patients. Four of the five studies reported poor functional outcomes in patients with higher BNP/NT-proBNP levels and one study associated higher BNP/NT-proBNP levels with increased risk of adverse cardiac events. Moreover, two studies identified an additional predictive ability of BNP/NT-proBNP level beyond that of pre-existing prognostic variables. In terms of disease severity, five studies (71%) reported that BNP/NT-proBNP level correlated positively with hematoma volume in addition to ICH and GCS scores.

**Conclusion:** Elevated BNP/NT-proBNP level is associated with increased risk of all-cause mortality, poor functional outcome, adverse cardiac events and disease severity in patients with ICH. Thus, BNP/NT-proBNP level is a promising prognostic indicator for ICH and also an effective marker of disease severity. Current evidence remains limited by the small number and high heterogeneity of included studies. Further appropriately designed, large-scale studies are required to confirm the current findings.

## Introduction

Intracerebral hemorrhage (ICH) is the second most frequent subtype of stroke and affects ~2 million people worldwide each year (1). ICH is a highly fatal and disabling event, with case fatality of about 40% at 1 month and 60% at 1 year, and functional independency rates of only 12–39% (2). In spite of continuing advances in medical technology, there are no interventions that are effective in improving clinical outcomes after ICH. The early and accurate assessment of severity and prognosis is critical in allowing appropriate treatment decisions to be made for ICH patients. To date, several clinical, radiographic and laboratory parameters, such as the patient's age, state of consciousness and baseline hematoma volume (3–5), are acknowledged to be strong prognostic indicators following ICH. However, existing prognostic parameters have a limited ability for realistic evaluation of possible outcomes in an individual patient. It is possible to speculate that identification of appropriate blood biomarkers may enable a better prediction model.

Brain natriuretic peptide (BNP), a polypeptide neurohormone with natriuretic, diuretic and vasodilator properties, is synthesized and released primarily by ventricular myocytes (6, 7). Cleavage of BNP precursor (pro-BNP) releases BNP in addition to an inactive N-terminal fragment (NT-proBNP) (8), which has a longer half-life and higher blood concentration. Both peptides are secreted in response to increased ventricular load or wall tension (9). Both rise in concentration during the occurrence and development of cardiovascular disease and serve as valuable prognostic biomarkers, especially with respect to heart failure (9–11). In addition, there is growing evidence that plasma BNP/NT-proBNP levels are generally elevated in patients with acute brain injury (12, 13). High levels are associated with mortality and poor functional outcome after ischemic stroke and subarachnoid hemorrhage (14–16).

There is growing evidence that the brain interacts with the heart (17), that is, brain injury has an effect on the heart and vice versa. The activation of the neuroendocrine system (e.g., a significant increase in catecholamine levels) following ICH causes heart injury, thereby an increased level of BNP/NT-proBNP, which in return reflects the disease severity of ICH. In addition, cardiac dysfunction in ICH patients, often accompanied by elevated BNP/NT-proBNP level, might further aggravate the disease severity. Therefore, BNP/NT-proBNP may associated with the prognosis of ICH. Over the past decade, many studies have reported the correlation between BNP/NT-proBNP levels and clinical outcome of ICH patients (18–20). However, the role in assessment of mortality risk and poor functional outcome after ICH remains controversial. Indeed, there is considerable diversity among reports of the utility of BNP/NT-proBNP level for risk estimates. Furthermore, the prognostic value of BNP/NT-proBNP levels in ICH has not been methodically analyzed. To address these knowledge gaps, we conducted a systematic review of pre-existing studies. Our aim was to evaluate the association of BNP/NT-proBNP level with adverse clinical outcomes and disease severity in patients with ICH.

## Methods

### Search Strategy

The present systematic review was performed in accordance with the Preferred Reporting Items for Systematic Reviews and Meta-Analyses (PRISMA) statement (21). A literature search was conducted through four databases: PubMed, Embase, Web of Science and the Cochrane Library. Publication dates covered were between inception and July 23, 2021. MeSH terms used for the search were: “Brain Natriuretic Peptide,” “N-terminal pro-brain natriuretic peptide,” “Cerebral Hemorrhage,” and “Hemorrhagic Stroke.” Details of the search strategy are provided in Supplementary Method in Supplementary Material. In addition, the reference lists of eligible publications were manually retrieved to identify additional studies.

### Study Selection

Studies meeting the following criteria were included: (1) population: patients over 18 years old with ICH confirmed by computed tomography (CT); (2) exposure: levels of blood BNP or NT-proBNP; (3) comparison: cases with vs. without elevated BNP or NT-proBNP levels; (4) outcome measures: all-cause mortality, poor functional outcome, adverse cardiac events and markers of disease severity; (5) study design: longitudinal observational studies. Studies were excluded if the following conditions applied: (1) cerebral hemorrhage secondary to trauma, brain tumor or vascular malformation, hemorrhagic transformation of brain infarcts, aneurysm associated ICH; (2) a mixed population of patients were described (e.g., stroke), unless patients with ICH were reported separately within the publication; (3) conference abstracts, reviews or case reports; (4) repeated studies of the same patient population (only the study with the largest sample size or the most detailed information was included); (5) patients with heart disease were included but adjusted risk estimate was not reported.

Two independent authors made a preliminary examination of the titles and abstracts to exclude studies that were obviously irrelevant. The full texts of the remaining studies were then reviewed to determine the included studies according to the aforementioned inclusion and exclusion criteria. Disagreements were resolved by involving the third reviewer.

### Data Extraction

Data extraction was completed independently by two authors using standardized data extraction forms. The following information of each study was collected: the first author's surname, publication year, country of origin, study design, recruiting period, number of participants, mean ages of participants, percentage of male participants, intervals from onset to admission, type of natriuretic peptide, blood collection time, methods of BNP/NT-proBNP detection, levels of BNP/NT-proBNP (mean ± SD), outcome measures, follow-up time, outcomes [including odds ratio (OR), relative risk (RR) or hazard ratio (HR) with 95% confidence interval (CI)], cutoff values of BNP/NT-proBNP levels (sensitivity and specificity, if available) and the area under the receiver operator characteristic (ROC) curve (AUC), and any further findings relevant to this review. Any disagreements between the two authors were settled through involving the third reviewer. When data provided in an article were insufficient or missing, we attempted to contact the respective authors for further information.

### Outcomes

The primary objective of the current review was to produce a qualitative analysis of the correlation between BNP/NT-proBNP level and adverse clinical outcomes in ICH patients. The secondary objective was to evaluate the relationship between BNP/NT-proBNP level and disease severity. The primary endpoint was prognosis, including all-cause mortality, poor functional outcome (the primary prognostic indicators) and adverse cardiac events (the secondary prognostic indicator). Functional outcome was assessed using the modified Rankin Scale (mRS) or Glasgow Outcome Scale (GOS), in which an mRS score of >2 or a GOS score of ≤ 3 was defined as a poor functional outcome. Adverse cardiac events included cardiac death, severe arrhythmia (paroxysmal supraventricular tachycardia, atrial tachycardia, ventricular tachycardia, atrial flutter, atrial fibrillation and ventricular fibrillation) and non-fatal myocardial infarction. The secondary endpoint was disease severity, evaluated by hematoma volume (calculated using the ellipsoid volume equation based on CT scans) (22), Glasgow Coma Score (GCS), ICH score and intraventricular hemorrhage.

### Quality Assessment

The Newcastle-Ottawa Scale (NOS) for the cohort studies (23) was utilized to evaluate the quality of included studies. This scale incorporates three aspects: selection (0–4 points), comparability (0–2 points), and outcome (0–3 points), with a total score of 9. Studies achieving seven points or more were considered high quality (24). The quality assessment was performed by two independent authors with a third reviewer arbitrating in case of disagreement.

### Data Synthesis

A narrative summary of results was presented (qualitative analysis). The heterogeneity between the included studies precluded the application of data synthesis.

## Results

### Search Results

**Figure 1:**
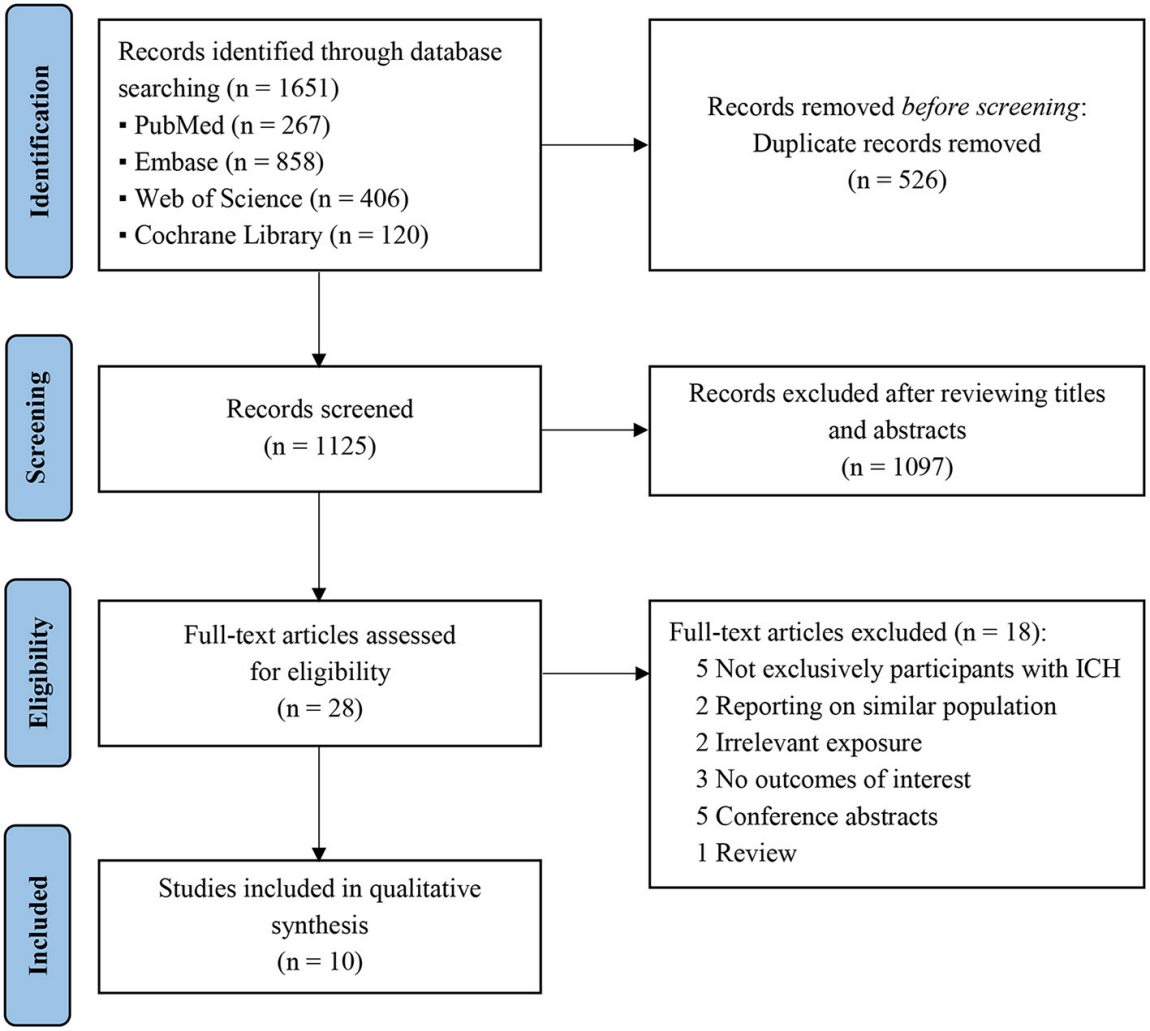
PRISMA flow diagram representing the search and selection process. ICH, intracerebral hemorrhage.

An electronic search retrieved a total of 1,651 articles with no additional articles being identified via a manual search. After eliminating duplications, 1,125 articles were retained, of which 1,097 were excluded due to irrelevant titles and abstracts. The full texts of the remaining 28 articles were reviewed for inclusion, and 10 studies (18–20, 25–31) fulfilled the inclusion criteria for systematic review. The detailed process of study screening is shown in Figure 1.

### Study Characteristics

Table 1 summarizes the main characteristics of included studies. Ten eligible studies all adopted a prospective design, of which two (19, 30) recruited participants from multiple centers. A total of 1,373 patients with ICH were included (28–271 subjects per study). All studies were restricted to ICH patients except one (29) involving both ICH (91 cases) and ischemic stroke patients in two separate cohorts. Mean ages of participants ranged from 60.7 to 72 years, and the percentage of males was 47–66%, with one study (29) not reporting gender distribution and two (25, 29) omitting age. Admission was within 24 h of ICH onset, except for two studies (20, 27) that did not record onset time. Collection of blood samples for BNP or NT-proBNP assay was within 24 h of admission, and four studies (19, 26, 29, 30) reported BNP/NT-proBNP levels at additional time points. Follow-up time varied from hospital discharge to 6 months after onset.

**Table 1:** Baseline characteristics of included studies.

**References**	**Country**	**Study design**	**Recruiting period**	**No. of participants**	**Onset to admission**	Age^*^ (years)	**Male**	**Biomarker/** **Level^#^ (pg/ml)**	**Blood collection time^&^**	**Detection method**	**Outcomes**
James et al. (25)	USA	Prospective	2000–2003	28	<24 h	-	50%	BNP	Within 24 h	FEIA	② ④
								236.4 ± 245.6		*Biosite*	
Goya et al. (18)	Japan	Prospective	2007–2011	271	<24 h	72^$^	60%	BNP	On admission	CLIA	①
								-		*Shionogi*	
Shibazaki et al. (26)	Japan	Prospective	2006–2010	250	<24 h	68.5	66%	BNP	On admission, and day 28	CLIA	① ④
								71.1 ± 104.1		*Shionogi*	
Park et al. (27)	Korea	Prospective	2009–2011	77	-	66	51%	BNP	On admission	FEIA	③
								295.6 ± 575.0		*Abbott*	
Li et al. (19)	China	Prospective	2015–2016	132	<8 h	50–74^†^	47%	NT-proBNP	On admission, and days 4, 7	ECLIA	① ②
								426.5 ± 224.1		*Roche*	
Niu and Teng (28)	China	Prospective	2015–2016	126	<24 h	63.8	60%	NT-proBNP	Within 24 h	ECLIA	① ④
								728.4 ± 143.9		*Roche*	
Yang et al. (29)	China	Prospective	2015–2017	91	<24 h	-	-	NT-proBNP	Within 24 h, and days 7, 14	ECI/ECiQ	② ④
								460.7 ± 296.8		*Ortho*	
Li et al. (30)	China	Prospective	2015–2016	147	<24 h	63	47%	NT-proBNP	On admission, and days 1–14	ECLIA	④
								372.5^‡^		*Roche*	
Gregorio et al. (20)	Portugal	Prospective	2012–2017	201	-	69.9	63%	NT-proBNP	On admission	ECLIA	① ② ④
								321.0 (118.0–859.5)		*Elecsys*	
Eldawoody et al. (31)	Egypt	Prospective	12-month	50	<24 h	60.7	64%	NT-proBNP	Within 24 h	ECLIA	② ④
								-		*Roche*	

*BNP, brain natriuretic peptide; NT-proBNP, N-terminal pro-brain natriuretic peptide; FEIA, fluorescent enzyme immunoassay; CLIA, chemiluminescence immunoassay; ECLIA, electrochemiluminescence immunoassay.*

① *, all-cause mortality;,*

② *functional outcome;,*

③ *adverse cardiac events;,*

④ *disease severity.*

* *Age was reported as mean, if not otherwise specified; the study used a*

$ *median or*

† *range to describe age.*

# *Admission BNP/NT-proBNP level, expressed as mean ± SD or median (interquartile range).*

& *Blood collection time was time since hospital admission.*

‡ *Mean NT-proBNP level was extracted from the images of the article*.

Nine studies involving 1,226 participants reported an association between BNP/NT-proBNP levels and adverse clinical outcomes and seven (893 participants) mentioned a relationship with disease severity. The NOS scores of the included studies ranged from 6 to 8, indicating moderate to high quality (Supplementary Table 1).

### BNP/NT-proBNP Levels in ICH Patients

Four studies under scrutiny (18, 25–27) focused on BNP and the remaining six on NT-proBNP. Most studies collected venous blood samples (four studies not specified) to detect plasma or serum BNP/NT-proBNP levels. Diverse methods for BNP/NT-proBNP measurement were employed with electrochemiluminescence immunoassay being the most frequently used (five studies; 50%). Chemiluminescence immunoassay (18, 26) and fluorescence enzyme immunoassay (25, 27) were used in two studies each. One study (29) measured NT-proBNP level by intellicheck technique using Vitros ECI/ECiQ (Ortho Clinical Diagnosis, USA). The baseline (admission) BNP/NT-proBNP levels of ICH patients also varied significantly among studies, ranging from 71.1 to 728.4 pg/ml.

Four studies evaluated BNP/NT-proBNP concentration changes with time. Li et al. (30) conducted continuous measurement of plasma NT-proBNP levels for 14 days after ICH onset. Their results revealed that the levels of NT-proBNP were elevated progressively and notably after ICH and reached a peak on day 4 (mean: 666.8 ± 355.1 pg/ml), and then gradually decreased up to day 14. Similarly, another study (19) showed that plasma NT-proBNP levels rose to a peak on day 4 (mean: 745.95 ± 428.89 pg/ml) before declining by day 7 to reach a value that was higher than that on day 1. It should be noted that both studies were reported by the same authors. However, Yang et al. (29) compared the levels of serum NT-proBNP with the improvement of symptoms, finding an inverse correlation (levels on day 1 > day 7 > day 14; *P* < 0.01). Shibazaki et al. (26) also found higher plasma BNP levels in the acute phase of ICH compared with those in the subacute phase (mean values from 4 h and 4 weeks after ICH onset: 69.3 ± 108.1 vs. 21.7 ± 23.5 pg/ml; *P* < 0.0001).

### Association of BNP/NT-proBNP Levels With the Prognosis of ICH

#### All-Cause Mortality

Table 2 summarizes the association between BNP/NT-proBNP levels and prognosis of ICH. Five studies (18–20, 26, 28) including 980 participants provided the data on all-cause mortality outcome, indicating a correlation with BNP/NT-proBNP level. Among them, four studies (18, 19, 26, 28) found consistently higher levels of BNP/NT-proBNP in non-survivors compared with survivors (range of means: 99.9–1986.48 vs. 32.4–432.38 pg/ml; *P* < 0.05). Three studies reported multivariate logistic regression analysis to assess risk estimate. Two studies correlated BNP/NT-proBNP levels on admission with the risk of short-term mortality. For in-hospital mortality (20), the OR of NT-proBNP level was 1.650 (95% CI 1.043-2.612; *P* = 0.032) after adjustment for covariates (e.g., age, gender and hematoma size). For 1-month mortality (18), the OR of BNP level was 4.7 (95% CI 1.43–15.63; *P* = 0.011). Another study (19) followed patients for 6 months to analyze the relationship between NT-proBNP level and long-term mortality. However, it should be noted in this study that although the serum NT-proBNP levels on days 1, 4, and 7 were all correlated with mortality after ICH, only that on day 4 (peak value) was an independent prognostic indicator of mortality (OR 1.004; 95% CI 1.001–1.007; *P* = 0.007). The series of studies outlined above indicated that higher BNP/NT-proBNP level was associated with increased risk of death after ICH. Thus, BNP/NT-proBNP level may have utility in predicting all-cause mortality for ICH patients.

**Table 2:** The association between BNP/NT-proBNP levels and prognosis of ICH.

**References**	**Outcome measure**	**Follow-up**	**No. of patients**	**OR (95% CI)**	***P*** **-value**	**Statistical/** **Adjusted** **factors**	**Cutoff value (pg/ml)**	**Sensitivity/ Specificity**	**AUC**	**BNP/NT-proBNP level^#^**	
										**I (pg/ml)**	**II (pg/ml)**
**Mortality**											
Goya et al. (18)	All-cause mortality	1 month	271	4.7 (1.43–15.63)	0.011	MV/1, 2, 3	BNP: 60.0	69/67%	-	32.4 (17.3–85.0)	102.5 (48.7–205.0)^*^
Shibazaki et al. (26)	All-cause mortality	In-hospital	250	-	-	-	BNP: -	-	-	67.4 ± 104.1	99.9 ± 101.6^*^
Li et al. (19)	All-cause mortality	6 months	132	1.004 (1.001–1.007)	0.007	MV^†^	NT-proBNP: 999.85	93.8/92.0%	0.958	332.88 ± 141.28	719.08 ± 178.45^*^
Niu and Teng (28)	All-cause mortality	6 months	126	-	-	-	NT-proBNP:-	-	-	432.38 ± 183.26	1986.48 ± 450.73^*^
Gregorio et al. (20)	All-cause mortality	In-hospital	201	1.650 (1.043–2.612)	0.032	MV/1, 2, 3	NT-proBNP: -	-	-	-	-
**Functional outcome**											
James et al. (25)	Poor functional outcome: mRS > 2	Hospital discharge	28	1.023 (1.002–1.044)	0.04	UV/NA	BNP: -	-	-	-	-
Li et al. (19)	Poor functional outcome: GOS ≤ 3	Hospital discharge	132	1.004 (1.001–1.006)	0.008	MV^†^	NT-proBNP: 999.85	66.1/98.7%	0.838	302.09 ± 81.37	595.35 ± 245.29^*^
Yang et al. (29)	Functional outcome: mRS 0–5^&^	3 months	91	-	-	-	NT-proBNP: -	-	-	430.47 ± 109.63^&^	859.64 ± 128.56^&^^*^
Gregorio et al. (20)	Poor functional outcome: mRS > 2	3 months	193	1.449 (1.106–2.034)	0.009	MV/1, 2, 3	NT-proBNP: -	-	-	-	-
Eldawoody et al. (31)	Poor functional outcome: mRS^$^	Hospital discharge	50	-	0.4	MV/NM	NT-proBNP: -	-	-	-	-
**Adverse cardiac events**											
Park et al. (27)	Adverse cardiac events	In-hospital	77	1.003 (1.001–1.005)	0.01	MV^†^	BNP: 156.6	68/66%	0.749	168.5 ± 173.5	683.8 ± 1043.8^*^

*BNP, brain natriuretic peptide; NT-proBNP, N-terminal pro-brain natriuretic peptide; ICH, intracerebral hemorrhage; OR, odds ratio; CI, confidence interval; AUC, area under the curve; MV, multivariate logistic regression; UV, univariate logistic regression; mRS, modified Rankin Scale; GOS, Glasgow Outcome Score; NA, not applicable; NM, not mentioned.*

*We record “1” for study which adjusted for heart disease including coronary heart disease, congestive heart failure, and atrial fibrillation; “2” adjusted for antiplatelet treatment; “3” adjusted for anticoagulation therapy.*

*Mortality outcome: event I represented survival and event II represented death; functional outcome: events I and II represented good and poor functional outcome, respectively; adverse cardiac events: events I and II represented absence and presence of cardiac events, respectively.*

* *There was a statistical difference in BNP/NT-proBNP level between event I and event II (P < 0.05).*

# *BNP/NT-proBNP level was presented as mean ± SD or median (interquartile range).*

& *In this study, the mRS was evaluated as a continuous variable (scores of 0–5), and good or poor functional outcome was not defined. Event I represented an mRS score of 2; event II represented an mRS score of 4.*

$ *In this study, the mRS was evaluated as a dichotomous variable, but the specific mRS scores for poor functional outcome were not defined.*

† *This study did not include patients with a history of heart disease including congestive heart failure, coronary heart disease and atrial fibrillation*.

Two studies reported ROC curves to evaluate the efficiency of BNP/NT-proBNP levels in discriminating between death and survival outcomes. Goya et al. (18) demonstrated an optimal cutoff BNP concentration in predicting mortality of 60.0 pg/ml giving 69.0% sensitivity and 67.0% specificity. Case fatality rates of patients with BNP levels > and ≤ 60.0 pg/ml were 31.1 and 13.5%, respectively. And Li et al. (19) established an NT-proBNP cutoff concentration of 999.85 pg/ml, with the sensitivity and specificity of 93.8 and 92.0%, respectively. The AUC was 0.958. Furthermore, Gregorio et al. (20) reported the incremental value of NT-proBNP level on the ICH Grading Scale, the accepted prediction model for mortality.

#### Functional Outcome

Five studies (19, 20, 25, 29, 31) involving 494 participants reported the association between BNP/NT-proBNP level and functional outcome in patients with ICH, assessed using mRS (four studies) or GOS (one study). Yang et al. (29) treated the mRS score as a continuous variable rather than a dichotomous outcome (good vs. poor functional outcome). They compared the serum NT-proBNP levels among ICH patients with different mRS scores (0–5 points), and the results suggested that the higher the NT-proBNP levels on admission, the higher the 3-month mRS scores, that was, the poorer the functional prognosis. Another four studies presented multivariate (univariate) logistic regression analyses to assess the predictive value of BNP/NT-proBNP level for poor functional outcome. Among them, three studies showed that elevated BNP/NT-proBNP level was independently correlated with poor functional outcome after ICH. These three studies were all short-term follow-up (hospital discharge or 3 months). One study (25) analyzed the association between BNP level and the risk of poor functional outcome (OR 1.023; 95% CI 1.002–1.044; *P* = 0.04), and two focused on NT-proBNP: Gregorio et al. (20) demonstrated an adjusted OR of 1.449 (95% CI 1.106–2.034; *P* = 0.009) for poor functional outcome after adjustment for age, gender, GCS score, hematoma size and Graeb score; and Li et al. (19) gave a OR of 1.004 (95% CI 1.001–1.006; *P* = 0.008). By contrast, Eldawoody et al. (31) indicated a non-significant correlation between NT-proBNP and functional outcome at discharge (*P* = 0.4; OR value not reported).

Only one study (19) reported a ROC curve for the predictive utility of NT-proBNP level with respect to poor functional outcome. The AUC was 0.838, and applying an NT-proBNP cutoff value of 999.85 pg/ml gave sensitivity and specificity of 66.1% and 98.7%, respectively. In this study, the NT-proBNP levels were also compared between the patients with good and poor functional outcomes, showing significant difference (302.09 ± 81.37 vs. 595.35 ± 245.29 pg/ml; *P* < 0.001). What's more, James et al. (25) and Gregorio et al. (20) separately included BNP and NT-proBNP levels into the traditional prognostic variables of ICH (such as FUNC and ICH score), both of which displayed added value for the prediction of functional outcome.

#### Adverse Cardiac Events

One study (27) reported that elevated serum BNP levels on admission were independently associated with in-hospital adverse cardiac events in patients with ICH (OR 1.003; 95% CI 1.001–1.005; *P* = 0.01). ROC curves analysis revealed that a cutoff BNP level of 156.6 pg/ml gave a sensitivity of 68% and a specificity of 66% for prediction of adverse cardiac events, with the AUC of 0.749.

### Association of BNP/NT-proBNP Levels With the Severity of ICH

As shown in Table 3, seven studies (20, 25, 26, 28–31) mentioned the relationship between BNP/NT-proBNP levels and disease severity in ICH patients. In these studies, the most commonly used marker to reflect disease severity was ICH volume (7 studies, 100%), assessed by CT scans on admission. Five studies (20, 26, 28–30) showed that BNP/NT-proBNP level was positively correlated with ICH volume. However, James et al. (25) and Eldawoody et al. (31) suggested a non-significant association between BNP/NT-proBNP levels and hematoma volume, although both indicated the relationship between BNP/NT-proBNP and ICH score. Three studies (28–30) divided patients into massive (>30 ml) and small-moderate (<30 ml) hemorrhage groups based on hematoma volume [Yang et al. (29) used 20 ml as a cutoff value], indicating higher levels of NT-proBNP in the former (range of means: 801.7-2116.3 vs. 165.5-493.7 pg/ml; *P* < 0.05). Two studies (29, 30) adopted the GCS score to evaluate disease severity, and both showed that NT-proBNP levels in patients with severe ICH were remarkably higher compared to mild-moderate patients (range of means: 912.4–939.6 vs. 262.8–454.0 pg/ml; *P* < 0.05), regardless of the inconsistent definition of the GCS scores for severe ICH (<11 and ≤ 8, respectively). Moreover, Niu and Teng (28) confirmed a correlation between NT-proBNP level and initial GCS score. Thus, it can be concluded that the larger the bleed or the more severe the disease, the higher the BNP/NT-proBNP level. BNP/NT-proBNP level may, therefore, be used to reflect the severity of ICH.

**Table 3:** The correlation between BNP/NT-proBNP levels and severity of ICH.

**References**	**Type of natriuretic peptide**	Markers of disease severity^*^	**No. of patients**	**Main findings**
James et al. (25)	BNP	ICH volume, GCS, ICH score, MLS	28	•BNP was not correlated with ICH volume (*r*^2^ = 0.14, *P* = 0.49) or GCS (*r*^2^ = 0.21). •BNP level was significantly associated with ICH score (*r*^2^ = 0.42, *P* = 0.02) and MLS (*r*^2^ = 0.42, *P* = 0.0002).
Shibazaki et al. (26)	BNP	ICH volume, hydrocephalus, intraventricular extension	250	•The log BNP level was positively associated with ICH volume (*r* = 0.132, *P* = 0.0376). •BNP was correlated with hydrocephalus (*P* = 0.0046) and intraventricular extension (*P* = 0.0039).
Niu and Teng (28)	NT-proBNP	ICH volume, GCS	126	•NT-proBNP levels: small < moderate < massive hemorrhage (*F* = 503.17, *P* < 0.001). [165.5 ± 58.4^#^ (ICHV <10 ml) vs. 417.3 ± 172.5 (10–30 ml) vs. 2116.3 ± 508.9 (>30 ml)] •NT-proBNP level correlated positively with ICH volume (*r* = 0.54, *P* < 0.001). •NT-proBNP was associated with GCS (*r* = 0.43, *P* = 0.001).
Yang et al. (29)	NT-proBNP	ICH volume, GCS, NIHSS	91	•NT-proBNP levels: small < moderate < massive hemorrhage (*F* = 47.92, *P* < 0.001). [246.1 ± 118.3^#^ (ICHV <10 ml) vs. 480.4 ± 201.3 (10–20 ml) vs. 801.7 ± 231.7 (>20 ml)] •NT-proBNP levels: mild < moderate < severe patients (*F* = 75.46, *P* < 0.001). [262.8 ± 130.7^#^ (GCS scores of 14–15) vs. 422.4 ± 178.9 (11–13) vs. 912.4 ± 181.3 (<11)] •NT-proBNP levels: mild < moderate < severe patients (*F* = 24.19, *P* < 0.01). [263.1 ± 129.6^#^ (NIHSS scores of 0–8) vs. 472.3 ± 265.9 (9–12) vs. 765.3 ± 291.3 (>12)]
Li et al. (30)	NT-proBNP	ICH volume, GCS, ICP, hyponatremia	147	•The level of NT-proBNP on day 4 after admission correlated positively with ICH volume (*r* = 0.702, *P* < 0.05). •Days 1–14^&^, NT-proBNP levels: small-moderate < massive hemorrhage (*P* < 0.05). [day 4: 493.7 ± 143.4^#^ (ICHV <30 ml) vs. 897.6 ± 417.8 (>30 ml)] •Days 1–14^&^, NT-proBNP levels: mild-moderate < severe patients (*P* < 0.05). [day 4: peak-to-mean concentration: 454.0^#^ (GCS > 8) vs. 939.6 ± 421.7 (≤ 8)] •In patients with severe ICH (57 cases), NT-proBNP level was positively correlated with ICP (*r* = 0.703, *P* < 0.05), but negatively with blood sodium concentration (*r* = −0.704, *P* < 0.05), which did not occur in mild-moderate patients.
Gregorio et al. (20)	NT-proBNP	ICH volume, Graeb Score	201	•Ln (NT-proBNP) was positively associated with ICH volume (*r* = 0.186, *P* = 0.008) and Graeb Score (amount of IVH) (*r* = 0.240, *P* = 0.001).
Eldawoody et al. (31)	NT-proBNP	ICH volume, ICH score, IVH	50	•NT-proBNP was not correlated with ICH volume (*P* = 0.6). •NT-proBNP level was significantly associated with ICH score (*P* = 0.04). •NT-proBNP levels: patients without IVH < patients with IVH (*P* = 0.02). [46.1 (5.9–2428) ^#^ vs. 243 (72.7–2994)]

*BNP, brain natriuretic peptide; NT-proBNP, N-terminal pro-brain natriuretic peptide; ICH, intracerebral hemorrhage; GCS, Glasgow Coma Score; MLS, midline shift; ICHV, hematoma volume; IVH, intraventricular hemorrhage; NIHSS, National Institute of Health stroke scale; ICP, intracranial pressure.*

* *These markers of disease severity were assessed on admission.*

# *BNP/NT-proBNP level (pg/ml) was presented as mean ± SD or median (interquartile range).*

& *Days 1–14 after admission*.

In addition, Li et al. (30) evaluated the relationship between NT-proBNP level and intracranial pressure and hyponatremia after ICH. Their results revealed that in patients with severe ICH (GCS scores of ≤ 8), serum NT-proBNP level was positively associated with intracranial pressure (*r* = 0.703), but negatively with blood sodium concentration (*r* = −0.704), which did not occur in mild-moderate patients. Further studies demonstrated associations between BNP/NT-proBNP levels and intraventricular hemorrhage (20, 31), ventricular dilatation and hydrocephalus (26).

### Predictors of BNP/NT-proBNP Levels in ICH Patients

Three studies (18, 20, 26) described the factors that affected the levels of BNP/NT-proBNP in ICH patients, indicating that BNP/NT-proBNP levels were independently associated with demographic factors such as age and gender, as well as history of heart disease, systolic blood pressure, blood glucose, creatinine clearance, and anticoagulation therapy. Therefore, when BNP/NT-proBNP level is utilized to assess the prognosis after ICH, values should be corrected for the influence of the above factors.

## Discussion

In this systematic review, we comprehensively evaluated the levels of BNP/NT-proBNP with regard to prognosis and disease severity in ICH patients. We found an association of BNP/NT-proBNP levels with all-cause mortality, poor functional outcome and adverse cardiac events after ICH, and also with disease severity markers (e.g., hematoma volume, ICH score and GCS score). Our analysis indicates the utility of BNP/NT-proBNP levels as a potential biomarker for disease severity assessment and prognostic evaluation of ICH patients.

ICH accounts for half of the disability-adjusted life years lost due to stroke worldwide (32). Although the case-fatality rate of ICH has declined in recent decades, mortality in the acute phase has not improved (33). Besides, studies showed that most deaths and disabilities, with accompanying hospitalization costs, occurred during the first year after ICH (34), illustrating the necessity of early and effective treatment. Rapid assessment of the severity and prognosis following ICH would accelerate the identification of the best therapeutic schedule and deliver better outcomes to patients. In the past, a number of variables, such as age, GCS score and initial hematoma characteristics (location, size and presence of intraventricular hemorrhage), have been employed to predict clinical outcome of ICH (35) and to develop prognostic models (5, 36). Such variables and prognostic models are difficult to manage in clinical practice, which limits their utility in improving outcomes. In recent decades, biomarkers have shown great promise for diagnosis and prognostic evaluation of central nervous system diseases (37–39), and when used in combination with pre-existing imaging and clinical parameters, they may provide additional prognostic information to help guide early treatment decisions. Importantly, dynamic measurement of biomarkers represents a considerable advantage over continuous imaging for the monitoring of disease progression. The roles of a number of blood biomarkers in distinguishing between ischemic and hemorrhagic stroke (40, 41) and predicting hematoma expansion for ICH patients (42, 43) have been reported. However, few studies have focused on the potential for biomarkers in prognosis. BNP and NT-proBNP are two widely used cardiac biomarkers that constitute vital predictors of mortality in acute coronary syndrome, cardiomyopathy and heart failure (44–46). Their prognostic value in ischemic stroke (16) and subarachnoid hemorrhage (47) has also been identified. Previous studies have reported the correlation between BNP/NT-proBNP level and clinical outcome in ICH patients. However, these studies tended to be small in scale, making their conclusions controversial. Therefore, there remained a pressing need for a larger-scale systematic review to summarize the findings.

Our literature survey has revealed a consistent relationship between BNP/NT-proBNP levels and mortality, poor functional outcome and adverse cardiac events after ICH. Moreover, the predictive value was independent of previously recognized prognostic factors such as age, hematoma volume, and baseline neurological impairment. However, in terms of functional outcome, one (31) of the five included studies did not report the correlation between serum NT-proBNP level and poor functional outcome. This omission might be due to the high heterogeneous of the included patients (initial neurological function and hematoma size), limited sample size (50 cases) or short follow-up time (hospital discharge). What's more, two studies (20, 25) showed the additional predictive ability of BNP/NT-proBNP levels beyond accepted prognostic variables. However, this finding should be viewed with caution due to the small sample sizes and further high-quality and large-scale studies are necessary for verification. In addition, we also found a close association between BNP/NT-proBNP level and the severity of ICH, especially in terms of the amount of bleeding. Previous studies have noted that such markers of disease severity were important predictors of adverse clinical outcomes (48, 49), thereby indirectly supporting a prognostic role for BNP/NT-proBNP level. In consequence of our findings, we recommend aggressive treatment for ICH patients with elevated BNP/NT-proBNP levels. In the future, further clinical trials are needed to evaluate whether reducing BNP/NT-proBNP levels improves the prognosis of ICH patients.

Mechanisms underlying the association between elevated BNP/NT-proBNP levels and adverse prognosis after ICH remain unclear. The triggering factors for BNP/NT-proBNP release are the changes in ventricular load and wall tension. The activation of the neuroendocrine system and the change of hemodynamic factors following ICH directly cause an increase in ventricular wall tension (29), and the elevation of blood pressure may lead to increased ventricular load, which thereby stimulate the secretion of cardiogenic BNP/NT-proBNP. In addition, the release of toxics, such as red blood cell degradation products, following the rupture of cerebral blood vessels, as well as the secondary cerebral edema may also stimulate BNP/NT-proBNP secretion from the central nervous system (50). Both mechanisms contributed to elevated blood BNP/NT-proBNP levels in ICH patients. Previous studies have identified hemodynamic changes in the acute phase of ICH, such as increased systemic vascular resistance, left ventricular hypoactivity and decreased cardiac output (51), causing increased BNP/NT-proBNP levels to reflect the hemodynamic stress response (20). This sequence of events clearly links BNP/NT-proBNP levels with prognosis. Moreover, hormones, such as natriuretic peptide, may contribute to the pathophysiological process of nerve injury in the early stages of ICH. The consequence of these events is that higher levels of BNP/NT-proBNP will accompany larger hematoma volumes and more serious brain damage. The resulting increased possibility of secondary brain edema or ischemia-hypoxia will worsen prognosis. Taken together, it is reasonable to conclude that BNP/NT-proBNP level correlates with clinical outcome of ICH but the specific mechanisms remain to be investigated in future research.

There are a few limitations to the current systematic review. First and most important, we can't pool data across studies and conduct a meta-analysis. Quantitative analysis of the included studies was not performed due to the limited sample size, methodological heterogeneity (e.g., different functional assessment scales, natriuretic peptide detection methods and follow-up times), and limited summary outcomes (e.g., some studies did not report effect sizes and we were unsuccessful in collection of missing data). Second, publication bias may influence our conclusions, since we only reviewed published articles, which may exclude studies with negative outcomes. Of relevance to this point is that five studies retrieved were published only as conference abstracts, of which one reported negative finding (Supplementary Table 2). Third, the diversity of detection methods led to different BNP/NT-proBNP levels among studies meaning that we were unable to specify a cutoff value for prediction of death or poor functional prognosis, limiting the clinical usefulness of our inferences regarding this biomarker. In the future, well-designed diagnostic test studies are required to determine the cutoff value. Fourth, compared with BNP, NT-proBNP has a longer half-life, higher blood concentration, and more stable biological characteristics, making the clinical detection more convenient and sensitive. Future studies should analyze the two peptides separately and compare their predictive abilities. Besides, continuous measurement of serum BNP/NT-proBNP levels was performed in only one of the included articles (30), and further studies are needed to assess whether dynamic monitoring contributes to the risk stratification of ICH. Fifth, the included studies were characterized by restricted follow-up times with half only continuing to hospital discharge. Future studies should extend the follow-up times to expose the possibility of more patients achieving a good outcome during rehabilitation.

In conclusion, this systematic review presents evidence that the elevated levels of BNP/NT-proBNP are associated with higher all-cause mortality, increased risk of poor functional outcome and increased incidence of adverse cardiac events in patients with ICH. BNP/NT-proBNP level is thus an independent prognostic indicator for ICH patients and is also an effective marker of disease severity. However, the current findings are limited by the small number and high heterogeneity of included studies, and the clinical utility remains to be further confirmed. There is a need for further well-designed and large-scale cohort studies to replicate, expand and refine the current findings. Such studies will allow clarification of the predictive value of BNP/NT-proBNP levels for the prognosis of ICH.

## Data Availability Statement

The original contributions presented in the study are included in the article/Supplementary Material, further inquiries can be directed to the corresponding author/s.

## Author Contributions

PZ and ZT conceived and designed the study. JiaW, JinW, and PZ completed the publication screening, data extraction, and quality assessment of the eligible publications. JiaW drafted the manuscript. PZ reviewed and revised the manuscript. All authors have read and approved the final manuscript.

## Funding

This work was supported by the National Natural Science Foundation of China (Grant No. 82071330).

## Conflict of Interest

The authors declare that the research was conducted in the absence of any commercial or financial relationships that could be construed as a potential conflict of interest.

## Publisher's Note

All claims expressed in this article are solely those of the authors and do not necessarily represent those of their affiliated organizations, or those of the publisher, the editors and the reviewers. Any product that may be evaluated in this article, or claim that may be made by its manufacturer, is not guaranteed or endorsed by the publisher.
