# Supplementary material for: Association of Natriuretic Peptide With Adverse Outcomes and Disease Severity After Intracerebral Hemorrhage: A Systematic Review
Source: Front Neurol. 2021 Nov 15;12:775085. doi: 10.3389/fneur.2021.775085 (PMC8634096; doi:10.3389/fneur.2021.775085)
Supplement: Supplementary file 1 [file Data_Sheet_1.PDF]

Supplementary Material

Supplementary Method

Search strategy for each database

PubMed

#1 ((((brain natriuretic peptide[Title/Abstract]) OR (B-type natriuretic peptide[Title/Abstract])) OR (ventricular natriuretic peptide, B-type[Title/Abstract])) OR (natriuretic factor 32[Title/Abstract])) OR (BNP[Title/Abstract])

#2 "Natriuretic Peptide, Brain"[Mesh]

#3 ((((((((N-terminal pro-brain natriuretic peptide[Title/Abstract]) OR (amino-terminal pro-brain natriuretic peptide[Title/Abstract])) OR (aminoterminal pro-B-type natriuretic peptide[Title/Abstract])) OR (pro-brain natriuretic peptide[Title/Abstract])) OR (N-terminal pro-BNP[Title/Abstract])) OR (NT-proBNP[Title/Abstract])) OR (NTproBNP[Title/Abstract])) OR (NT-BNP[Title/Abstract])) OR (N-BNP peptide[Title/Abstract])) OR (proBNP[Title/Abstract])

#4 "pro-brain natriuretic peptide (1-76)" [Supplementary Concept]

#5 #1 OR #2 OR #3 OR #4

#6 "Cerebral Hemorrhage"[Mesh]

#7 "Intracranial Hemorrhages"[Mesh]

#8 ((((((((((cerebr*[Title/Abstract]) OR (intracranial[Title/Abstract])) OR (intracerebral[Title/Abstract])) OR (brain[Title/Abstract])) OR (infratentorial[Title/Abstract])) OR (supratentorial[Title/Abstract])) OR (basal gangli*[Title/Abstract])) OR (putaminal[Title/Abstract])) OR (putamen[Title/Abstract])) OR (posterior fossa[Title/Abstract])) OR (hemispher*[Title/Abstract])

#9 ((((hemorrhag*[Title/Abstract]) OR (haemorrhag*[Title/Abstract])) OR (bleed*[Title/Abstract])) OR (hematoma*[Title/Abstract])) OR (haematoma*[Title/Abstract])

#10 #8 AND #9

#11 ICH[Title/Abstract]

#12 "Hemorrhagic Stroke"[Mesh]

#13 (hemorrhag* stroke*[Title/Abstract]) OR (haemorrhag* stroke*[Title/Abstract])

#14 #6 OR #7 OR #10 OR #11 OR #12 OR #13

#15 #5 AND #14

Embase

#1 'brain natriuretic peptide'/exp

#2 'brain natriuretic peptide':ti,ab,kw OR 'b-type natriuretic peptide':ti,ab,kw OR 'ventricular natriuretic peptide, b-type':ti,ab,kw OR 'natriuretic factor 32':ti,ab,kw OR bnp:ti,ab,kw

#3 'amino terminal pro brain natriuretic peptide'/exp

#4 'n-terminal pro-brain natriuretic peptide':ti,ab,kw OR 'amino-terminal pro-brain natriuretic peptide':ti,ab,kw OR 'aminoterminal pro-b-type natriuretic peptide':ti,ab,kw OR 'pro-brain natriuretic peptide':ti,ab,kw OR 'n-terminal pro-bnp':ti,ab,kw OR 'nt probnp':ti,ab,kw OR ntprobnp:ti,ab,kw OR 'nt bnp':ti,ab,kw OR 'n-bnp peptide':ti,ab,kw OR probnp:ti,ab,kw

#5 #1 OR #2 OR #3 OR #4

#6 'brain hemorrhage'/exp

#7 cerebr*:ti,ab,kw OR intracranial:ti,ab,kw OR intracerebral:ti,ab,kw OR brain:ti,ab,kw OR infratentorial:ti,ab,kw OR supratentorial:ti,ab,kw OR 'basal gangli*':ti,ab,kw OR putaminal:ti,ab,kw OR putamen:ti,ab,kw OR 'posterior fossa':ti,ab,kw OR hemispher*:ti,ab,kw

#8 hemorrhag*:ti,ab,kw OR haemorrhag*:ti,ab,kw OR bleed*:ti,ab,kw OR hematoma*:ti,ab,kw OR haematoma*:ti,ab,kw

#9 #7 AND #8

#10 ich:ti,ab,kw

#11 'hemorrhag* stroke*':ti,ab,kw OR 'haemorrhag* stroke*':ti,ab,kw

#12 #6 OR #9 OR #10 OR #11

#13 #5 AND #12

Cochrane Library

#1 MeSH descriptor: [Natriuretic Peptide, Brain] explode all trees

#2 (brain natriuretic peptide):ti,ab,kw OR (B-type natriuretic peptide):ti,ab,kw OR (ventricular natriuretic peptide, B-type):ti,ab,kw OR (natriuretic factor 32):ti,ab,kw OR (BNP):ti,ab,kw 6304

#3 (N-terminal pro-brain natriuretic peptide):ti,ab,kw OR (amino-terminal pro-brain natriuretic peptide):ti,ab,kw OR (aminoterminal pro-B-type natriuretic peptide):ti,ab,kw OR (pro-brain natriuretic peptide):ti,ab,kw OR (N-terminal pro-BNP):ti,ab,kw OR (NT-proBNP):ti,ab,kw OR (NTproBNP):ti,ab,kw OR (NT-BNP):ti,ab,kw OR (N-BNP peptide):ti,ab,kw OR (proBNP):ti,ab,kw

#4 #1 OR #2 OR #3

#5 MeSH descriptor: [Cerebral Hemorrhage] explode all trees

#6 MeSH descriptor: [Intracranial Hemorrhages] explode all trees

#7 (cerebr*):ti,ab,kw OR (intracranial):ti,ab,kw OR (intracerebral):ti,ab,kw OR (brain):ti,ab,kw OR (infratentorial):ti,ab,kw OR (supratentorial):ti,ab,kw OR (basal gangli*):ti,ab,kw OR (putaminal):ti,ab,kw OR (putamen):ti,ab,kw OR (posterior fossa):ti,ab,kw OR (hemispher*):ti,ab,kw

#8 (hemorrhag*):ti,ab,kw OR (haemorrhag*):ti,ab,kw OR (bleed*):ti,ab,kw OR (hematoma*):ti,ab,kw OR (haematoma*):ti,ab,kw

#9 #7 AND #8

#10 MeSH descriptor: [Hemorrhagic Stroke] explode all trees

#11 (hemorrhag* stroke*):ti,ab,kw OR (haemorrhag* stroke*):ti,ab,kw OR (ICH):ti,ab,kw

#12 #5 OR #6 OR #9 OR #10 OR #11

#13 #4 AND #12

Web of Science

#1 TS=(brain natriuretic peptide) OR TS=(B-type natriuretic peptide) OR TS=(ventricular natriuretic peptide, B-type) OR TS=(natriuretic factor 32) OR TS=(BNP)

#2 TS=(N-terminal pro-brain natriuretic peptide) OR TS=(amino-terminal pro-brain natriuretic peptide) OR TS=(aminoterminal pro-B-type natriuretic peptide) OR TS=(pro-brain natriuretic peptide) OR TS=(N-terminal pro-BNP) OR TS=(NT-proBNP) OR TS=(NTproBNP) OR TS=(NT-BNP) OR TS=(N-BNP peptide) OR TS=(proBNP)

#3 (#1) OR #2

#4 TS=(cerebr*) OR TS=(intracranial) OR TS=(intracerebral) OR TS=(brain) OR TS=(infratentorial) OR TS=(supratentorial) OR TS=(basal gangli*) OR

TS=(putaminal) OR TS=(putamen) OR TS=(posterior fossa) OR TS=(hemispher*)

#5 TS=(hemorrhag*) OR TS=(haemorrhag*) OR TS=(bleed*) OR TS=(hematoma*) OR TS=(haematoma*)

#6 (#4) AND #5

#7 TS=(hemorrhag* stroke*) OR TS=(haemorrhag* stroke*) OR TS=(ICH)

#8 (#6) OR #7

#9 (#3) AND #8
