# Supplementary material for: Association of Natriuretic Peptide With Adverse Outcomes and Disease Severity After Intracerebral Hemorrhage: A Systematic Review
Source: Front Neurol. 2021 Nov 15;12:775085. doi: 10.3389/fneur.2021.775085 (PMC8634096; doi:10.3389/fneur.2021.775085)
Supplement: Supplementary file 2 [file Data_Sheet_2.PDF]

Supplementary Material

Supplementary Tables

Supplementary Table 1. Quality assessment of the included studies using the Newcastle-Ottawa Scale

Reference	Selection				Comparability	Outcome			Score
	A	B	C	D	E	F	G	H	
James, 2019 (24)	★	★	★	★	★	★		★	7
Goya, 2014 (17)	★	★	★	★	★★	★		★	8
Shibazaki, 2014 (25)	★	★	★	★		★		★	6
Park, 2015 (26)	★	★	★	★	★★	★		★	8
Li, 2017 (18)	★	★	★	★	★			★	6
Niu, 2017 (27)	★	★	★	★			★	★	6
Yang, 2018 (28)	★	★	★	★			★	★	6
Li, 2018 (29)	★	★	★	★			★	★	6
Gregorio, 2019 (19)	★	★	★	★	★★	★		★	8
Eldawoody, 2020 (30)	★	★	★	★	★	★		★	7

A, representativeness of the exposed cohort. B, selection of the non-exposed cohort. C, ascertainment of exposure. D, demonstration that outcome of interest was not present at start of study. E, comparability of cohorts based on the design or analysis. F, assessment of outcome. G, follow-up long enough for outcomes to occur. H, adequacy of follow-up of cohorts.

Supplementary Table 2. Results of conference abstracts: the association between BNP/NT-proBNP levels and prognosis of ICH

Reference	Country	No. of patients	Biomarker/ Blood collection time	Outcome measures	Follow-up	OR (95% CI), P-value	Statistical analysis	Cutoff value	BNP related to prognosis
Blanco, 2011 (1)	Spain	134	pro-BNP/ on admission	poor functional outcome: mRS > 3	3 months<br>6 months<br>1 year	4.718 (1.113-20.001)<br>7.215 (1.030-50.518)<br>9.389 (1.274-69.178)	–	450 pg/ml<br>AUC: 0.770<br>sensitivity: 75.6%<br>specificity: 70%	Yes
	Pro-BNP level: poor functional outcome > good outcome (3480±8736 vs 504±799 pg/ml; P = 0.013).								
Goya, 2013 (2)	Japan	304	BNP/ on admission	mortality	1 year	2.9 (1.457-5.931), P = 0.0026	MV	50 pg/ml<br>–	Yes
	Median age: 72; male: 63%; baseline BNP level: 123.1±320.8 pg/ml.								
Prada, 2015 (3)	Spain	103	BNP/ on admission	mortality	–	1.1 (1.1-1.2), P = 0.02	MV	–	Yes
	Median age: 61.8; male: 66%; BNP level: death > survival (223.5±318.5 vs 82.4±95.6 pg/ml; P < 0.05).								
Radhakrish-nan, 2018 (4)	India	60	BNP/ –	mortality	in-hospital	–	MV	–	Yes
	Method of BNP detection: ELISA.								
Rotzel, 2019 (5)	Spain	120	BNP/ on admission	1. mortality<br>2. poor functional outcome: mRS > 2	–	–	UV	–	1. Yes<br>2. No
	Mean age: 63; male: 63%; BNP level: death > survival (199.1±37 vs 84.3±10 pg/ml; P = 0.005).								

BNP, brain natriuretic peptide; NT-proBNP, N-terminal pro-brain natriuretic peptide; ICH, intracerebral hemorrhage; OR, odds ratio; CI, confidence interval; mRS, modified Rankin Scale; AUC, area under the curve; MV, multivariate logistic regression; UV, univariate logistic regression; ELISA, enzyme-linked immunosorbent assay.
BNP/NT-proBNP level was presented as mean ± SD.

References

1. Blanco M, Arias S, Brea D, Rodriguez-Gonzalez R, Costa E, Sobrino T, et al. High PRO-BNP levels at admission are associated with poor long-term functional outcome in patients with intracerebral hemorrhage. *Cerebrovasc Dis.* (2011) 31:154. doi: 10.1159/000329448
2. Goya Y. Brain natriuretic peptide upon admission should predict patients mortality within 1 year after onset of intracerebral hemorrhage. *Stroke.* (2013) 44.
3. Aguillón Prada DL, Serrano Lázaro A, Huerta Bravo RC, Mesejo Arizmendi A, Segura Roca JM, Sanchis Miñana JC, et al. Brain injury biomarkers and inflammatory markers like pronostic factors on mortality in patients with spontaneous intracranial hemorrhage (medical-adults intensive care). *Intensive Care Medicine Experimental.* (2015) 3. doi: 10.1186/2197-425X-3-S1-A852
4. Radhakrishnan DM, Warrier A, Bhatia R, Bali P, Sisodia P, Gupta A, et al. Role of blood biomarkers in predicting outcomes after acute stroke. *Neurology.* (2018) 90.
5. Rotzel HB, Serrano Lazaro A, Blasco Cortes ML, Tellez TA, Mulet Mascarell S, Sanchis Piqueras C, et al. Brain injury biomarkers as prognostic factors in patients with spontaneous intracerebral hemorrhage. *Intensive Care Medicine Experimental.* (2019) 7. doi: 10.1186/s40635-019-0265-y
